# Tuberculosis burden in China: a high prevalence of pulmonary tuberculosis in household contacts with and without symptoms

**DOI:** 10.1186/1471-2334-14-64

**Published:** 2014-02-06

**Authors:** Zhongwei Jia, Shiming Cheng, Yan Ma, Tianhao Zhang, Liqiong Bai, Weiguo Xu, Xiaoxin He, Peiru Zhang, Jinkou Zhao, David C Christiani

**Affiliations:** 1 National Institute of Drug Dependence, Peking University, Beijing 100191, China; 2 Takemi Program in International Health, Department of Global Health and Population, Harvard School of Public Health, Boston, MA, USA; 3 National Center for Tuberculosis Control and Prevention, Chinese Center for Disease Control and Prevention, Beijing 100017, China; 4 Center office, Beijing Tuberculosis and Thoracic Tumor Research Institute, Beijing 101149, China; 5 Beijing Research Institute for Tuberculosis Control, Beijing 100035, China; 6 Hunan institute for Tuberculosis Control, Changsha 410013, China; 7 Jiangsu Center for Disease Control and Prevention, Nanjing 210009, China; 8 Sichuan Center for Disease Control and Prevention, Chengdu 610041, China; 9 Department of Environment health, Harvard School of Public Health, Boston, MA, USA

**Keywords:** Tuberculosis, Household contact, Index cases

## Abstract

**Background:**

In the context of decreasing tuberculosis prevalence in China, we examined the effectiveness of screening household contacts of tuberculosis patients.

**Methods:**

A tuberculosis survey was conducted in 2008. All 3,355 household contacts of notified tuberculosis cases were examined with a questionnaire interview, chest X-ray and three sputum smear tests. The effectiveness was examined by comparing the prevalence of pulmonary tuberculosis in household contacts with or without presenting clinical symptoms against the respective notification rates. Regression models were used to evaluate the factors associated with pulmonary tuberculosis.

**Results:**

Of the 3,355 household contacts, 92 members (2.7%) had pulmonary tuberculosis, among which 46 cases were asymptomatic. The prevalence of pulmonary tuberculosis and smear positive cases in household contacts without symptoms were 20 and 7 times higher than the notification rates in 2008, while those in household contacts with symptoms were 247 and 108 times higher than notification rates, respectively. The patients detected were mainly Index Cases’ spouses, sisters/brothers and those who were in contact with female Index Cases.

**Conclusions:**

The present study provides convincing evidence that household contacts of notified tuberculosis cases are at higher risk of developing tuberculosis. Routine screening for household contacts without any symptoms is recommended for sustained tuberculosis control in China as well as in the world.

## Background

A few studies have indicated that close contacts have a higher risk of developing active tuberculosis [1-5]. China national tuberculosis program has yet included case detection among this group due to inadequate resources in the past. By 2005, the coverage of DOTS has achieved 100% at county scale while the case detection rate and the cure rate of the new sputum smear positive patients reached 79%and 91% respectively in China, indicating that China met the WHO’s staggered global targets for detection and treatment of tuberculosis cases [6-9]. Given the nature of tuberculosis surveillance being passive, most patients could not be detected until they present symptoms and seek health care [10]. In 2007, China’s Ministry of Health piloted a new screening strategy among suspects in household contacts of notified tuberculosis cases (Index Cases) in several regions to inform a possible screening strategy nationwide [11]. More positive pulmonary tuberculosis patients have been detected in this pilot than routine surveillance [2], which inspired a more extensive case detection among all household contacts considering the Mega health investment in health medical reform to eliminate tuberculosis from population [12]. Since 2008, a rigid screening strategy in household contacts with symptoms has been tentatively extended to the household contacts without symptoms.

The present study examines effectiveness of the screening strategies among the household contacts with or without symptoms, to inform whether the screening strategy should cover the household contacts without symptoms.

## Methods

For the present study, the Index Case is defined as a smear positive pulmonary patient who was first to be diagnosed in the household, but not always the first real patient. To our knowledge, there is no consistent definition of household contacts [13]. In our study, the household contacts are either family members or not-family members who have been living in same house with the Index Case for more than two weeks in the past three months before the data when the Index Cases were diagnosed. Notified cases were the number of pulmonary tuberculosis cases registered in tuberculosis national surveillance system (TBNSS). We calculated notification rate by dividing the number of notified cases by the number of total population in a geographically defined location. The prevalence among household contacts was calculated as the percentage of pulmonary tuberculosis cases among 100,000 household contacts. Delayed reporting was defined as the interval being more than two weeks between the onset of any symptom and the presentation to a health care provider [14].

### Study settings and cases

**Figure 1:**
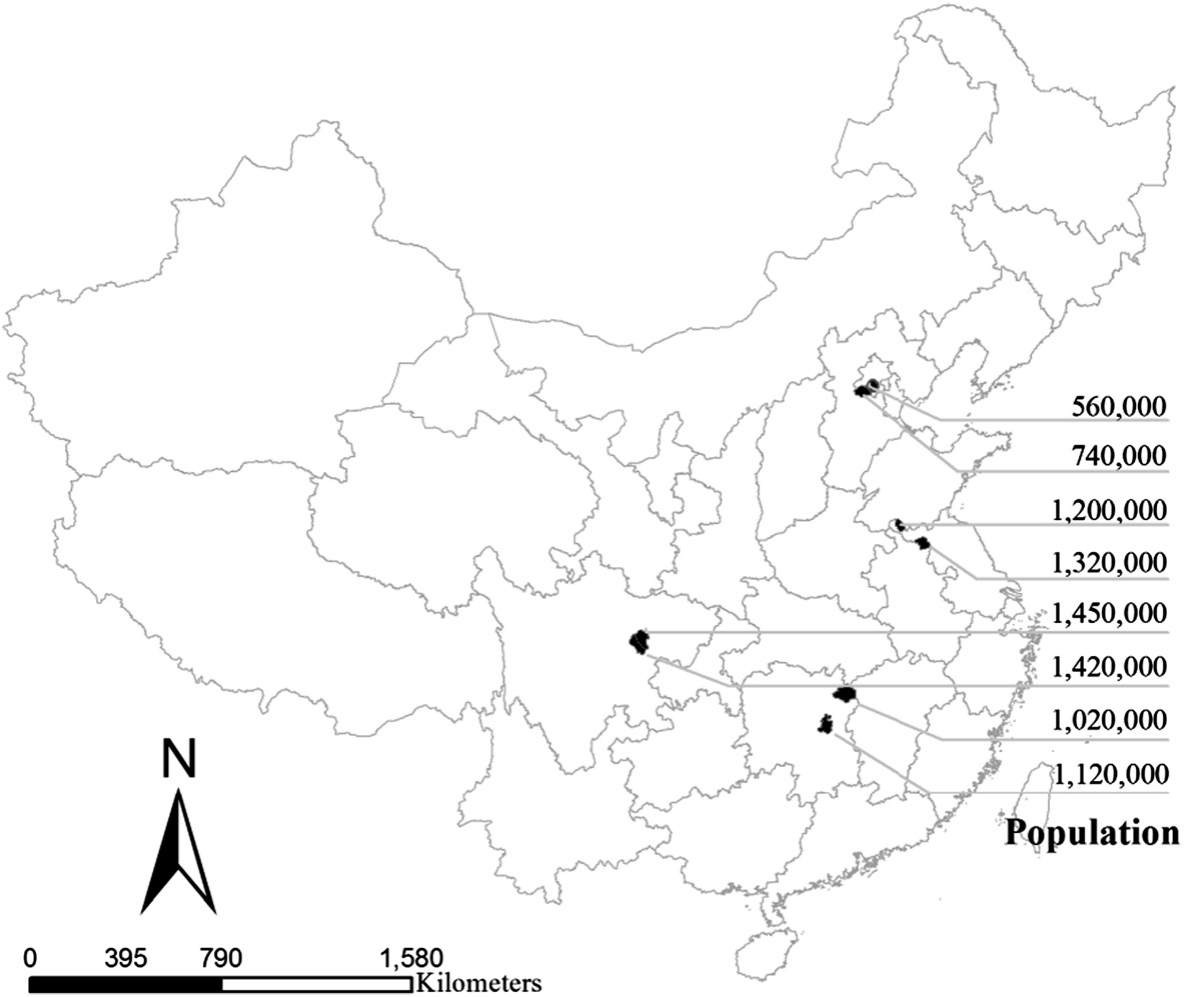
Location of study settings.

A cross-sectional survey was conducted among household contacts following the detection of the Index Case in the household in 2008. Based on the levels of tuberculosis burden in China [7], four regions, namely Eastern, Western, Central and Metropolitan regions were selected for the present study [7]. Two counties or urban districts in each region were selected, covering about 8.8 million populations (Figure 1). Since April, 1 2008, immediately after each of the Index Cases were diagnosed and notified by the local health professionals, all family members or relatives who have been living in same house with the Index Case for more than two weeks in the past three months before the data when the Index Cases were diagnosed (*household contacts*) were referred for a screening. All household contacts were administered a questionnaire interview by local health professionals, a chest X-ray examination at the local health facility, three sputum smear tests (the previous evening, the morning and at the moment of the day when seeing the local health professionals) were carried out at the county reference laboratories.

The demographic data, such as age, sex, have been gathered for both Index Cases and household contacts. Additional information were collected for the Index Cases, including education, whether or not re-treated cases, whether or not cavitary pulmonary tuberculosis, the onset of symptoms and the date of the first visit to local health facility. Additional information were also collected for household contacts, including whether having any symptoms, whether living in the same bedroom and the relationships with the Index Cases.

The primary outcome measurement is the prevalence pulmonary tuberculosis among the household contacts by whether having any symptoms while the secondary measurement is the effectiveness of the screening strategy among contacts without presenting any symptoms. All tuberculosis cases were diagnosed according to the latest guidelines issued by China Ministry of Health in 2003 [15]. For a diagnosis of smear-positive tuberculosis, one of three sets of conditions should be met: (1) 2 positive sputum smears by microscopy, (2) 1 positive sputum smear and 1 positive sputum culture, or (3) 1positive sputum smear-positive with typical pathology of active tuberculosis on a chest X-ray [15]. For a diagnosis of sputum smear-negative TB, the expert groups based on results of chest X-ray, purified protein derivative (PPD) and clinical manifestations, one of four conditions should be met after three negative sputum smear and 1 negative sputum culture: (1) typical pathology of active tuberculosis on a chest X-ray following symptoms such as cough, haemoptysis and fever, (2) typical pathology of active tuberculosis on a chest X-ray and diameter of PPD > 20 mm, (3) typical pathology of active tuberculosis on a chest X-ray and pathological changes of tuberculosis in extra-pulmonary tissues, (4) excluding other pulmonary diseases after following up or anti TB treatment for suspicious cases for three weeks. Active pulmonary tuberculosis cases include either smear positive or negative pulmonary tuberculosis cases [15].

### Ethical issues

This study was approved by the Chinese Ethical Committee for Tuberculosis Operational Research, Chinese Center for Disease Control and Prevention. A written informed consent was obtained from each of all participants prior to the interview.

### Statistical analysis

We compare the prevalence of pulmonary tuberculosis in household contacts who have presented symptoms to those who have not yet presented symptoms to examine the difference. We also compare the prevalence of pulmonary tuberculosis in the contacts with or without symptoms to the notification rate.

The factors associated with the transmission of pulmonary tuberculosis were examined by using a multilevel logistic saturated regression with the clinical diagnosis of pulmonary tuberculosis as an outcome. Independent variables included three kinds of variables. The first kind was characteristics of the index patients: age (0-14, 15-64 and >65), sex (0: women, 1: men), cavitary disease (yes:1, no: 0) , delay (yes:1, no :0) and treatment history (new: 0, retreatment: 1). The second kind was characteristics of the household contacts: age (0-14, 15-64 and >65), sex (0: women, 1: men) and symptoms (yes: 1, no: 0). The third kind was links between index patients and their contacts: relationship (spouse, parents, children, sister/brother and other), whether living same bedroom for index patients (yes: 1, no: 0). In the model, the household was fitted as 2-level and each contact as 1 level. Relative risks were calculated as crude odds ratios and adjusted odds ratios with 95% confidence intervals (CI) and a P value below0.05 indicates a statistical significance. All data were double entered using EpiData and were analyzedusingMLwiN2.02 and SPSS 17.

## Results

**Figure 2:**
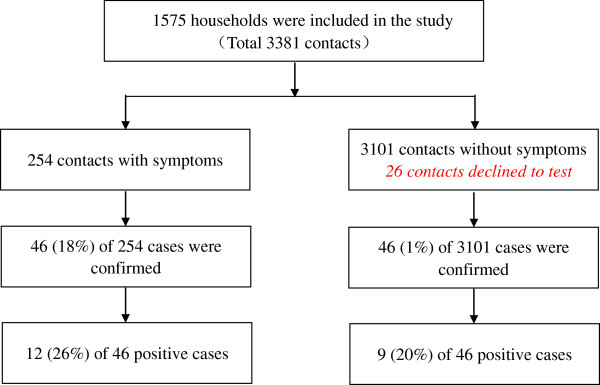
Summary of screening process.

Out of 1,575 households of Index Cases, 3,381 household contacts were identified and 3,355 (99.2%) examined (Figure 2). Most Index Cases (1,412/1,575) were the new positive cases, 77% were men and 80% were reported more than two weeks (delayed reporting) (Table 1).

**Table 1:** Occurrence of tuberculosis among the household contacts with and without symptoms by regions in 2008

	**Index patients**	**Overall**				**With symptoms**			**Without symptoms**		
		**Case**	**Contact**	**Prevalence**	**Notification**	**Case**	**Contact**	**Prevalence**	**Case**	**Contact**	**Prevalence**
				**1/100,000**	**1/100,000**			**1/100,000**			**1/100,000**
Overall	1575	92	3355	2742	**73.33**	46	254	18110	46	3101	1483
Positive cases	1575	21	3355	626	43.94	12	254	4724	9	3101	290
By regions											
Mega polis	175	2	363	551	21.77	1	15	6667	1	348	287
Centre region	437	26	661	3933	77.22	9	46	19565	17	615	2764
Eastern region	465	58	1051	5519	81.05	34	150	22667	24	901	2664
Western region	498	6	1280	469	79.93	2	43	4651	4	1237	323

Index patient: the first positive patient was found in family.

Case: the active pulmonary tuberculosis was found among the household contacts.

Contact: the number of family members who have contacted with the index patients.

Positive cases: the smear positive patients were found among the household contacts.

By regions: we divided whole country into 4 regions based on the distribution of TB in China.

Of the 3,355 household contacts, 92 people had pulmonary tuberculosis (2,742 per 100,000 populations) with 21 being smear positive cases (626 per 100,000 populations). Of 92 pulmonary tuberculosis diagnosed, 46 cases (9 smear positive cases) had not yet presented any clinical symptoms (Table 1). For household contacts without symptom, the prevalence of pulmonary tuberculosis (1,483 per 100,000 populations) was 20 times higher than the notified rate (73 per 100,000 populations) and the prevalence of smear positive cases (290 per 100,000 populations) was 7 times higher than the notification rate (43 per 100,000 populations). Among household contacts with symptoms, the prevalence of pulmonary tuberculosis and smear positive cases were 248 (18,110 per 100,000 populations) and 107 (4,724 per 100,000 populations) times higher than the notification rates respectively (Table 1).

By adjusting household clustering, household contacts presenting symptoms had 14 times higher risk to be diagnosed as pulmonary tuberculosis in comparison with the those who have not yet presented any symptoms (OR13.94, P < 0.05) (Table 2). The pulmonary tuberculosis was more likely to be found among the spouses or sisters/brothers of the Index Cases and among those who were in contact with the female Index Cases (Table 2). However, the pulmonary tuberculosis was not found associated with age, cavitary lesions, treatment history of the Index Cases and living in the same bedroom with the Index Cases tuberculosis (Table 2). Among household contacts, the elder people (aged above 65 years old) (AOR4.96, P < 0.05) and men (AOR1.52, P < 0.05) were most likely to be diagnosed tuberculosis (Table 2).

**Table 2:** Factors related with occurrence of tuberculosis in household contacts

	Number of IC*	**Number of contacts**	**Cases in contacts**	**Prevalence (1/100,000)**	**OR**	**AOR†**
Index patients						
Age						
0-14	7	12	1	8333	1.00	1.00
15-64	1224	2568	69	2687	0.31 (0.03, 3.48)	0.40 (0.03, 1.50)
65-	334	773	22	2846	0.33 (0.03, 3.87)	0.46 (0.04, 5.40)
Sex						
Woman	434	967	37	3826	1.00	1.00
Man	1141	2388	55	2303	0.59 (0.37, 0.94)‡	0.60 (0.36, 0.99)‡
Cavitary disease						
No	1153	2471	66	2671	1.00	1.00
Yes	414	868	26	2995	1.12 (0.67, 1.86)	1.22 (0.72, 2.07)
Treatment history						
New	1427	3089	80	2590	1.00	1.00
Re-treatment	144	258	12	4651	1.75 (0.80, 3.38)	1.65 (0.45, 3.23)
Treatment delay						
No	304	698	11	1576	1.00	1.00
Yes	1217	2657	81	3049	1.91 (0.98, 3.73)	1.70 (0.85, 3.71)
Household contacts						
Age						
0-14		359	4	1114	1.00	1.00
15-64		2649	70	2643	2.28 (0.83, 6.28)	2.30 (0.84, 6.30)
65-		342	18	5263	4.77 (1.59, 14.37)‡	4.96 (1.85, 14.89)‡
Sex						
Woman		1795	42	2340	1.00	1.00
Man		1560	50	3205	1.40 (0.91, 2.14)‡	1.52 (1.18, 2.32)‡
Symptoms						
No		3101	46	1483	1.00	1.00
Yes		254	46	18110	14.10 (8.91,22.31)‡	13.94 (8.61,12.59)‡
Index patients and household contacts						
Relationship						
Couple		949	39	4110	1.00	1.00
Parent		654	20	3058	0.73 (0.41, 1.29)	0.55 (0.20, 1.49)
Children		939	18	1917	0.44 (0.24, 0.79)‡	0.35 (0.12, 0.92)‡
Sister/brother		242	10	4132	0.86 (0.39, 1.89)	0.72 (0.23, 2.28)
Other		544	5	921	0.22 (0.08, 0.56)‡	0.40 (0.21, 0.77)‡
Whether living same bedroom						
No		2368	55	2323	1.00	1.00
Yes		987	37	3749	1.70 (1.09, 2.63)‡	1.35 (0.74, 2.50)
Random effect						
Constant						3.08 (1.05, 9.09)‡

Remark: * IC is Index Cases. †AOR is adjusted odds ratios. ‡ indicate 95% significant.

## Discussion

The study presents very high prevalence of pulmonary tuberculosis in the household contacts with or without any symptoms, which are about six times or nine times higher in comparison with report of 2010 national tuberculosis prevalence survey (2742 per 100,000 populations vs. 459 per 100,000 populations and 626 per 100,000 populations vs. 66 per 100,000 populations) respectively [16]. Assuming the difference in prevalence between the present study and 2010 national tuberculosis survey can be extrapolated to nationwide, there might have been 30,748 undiagnosed pulmonary tuberculosis cases nationwide if all the household contacts of all notified cases in 2008 were screened. This presents a great missed opportunity to detect a large pool of pulmonary tuberculosis in the population to further advance the remarkable achievement in past two decades with fighting against tuberculosis in China [9].

Not surprising, our study find that the household contacts presenting symptoms have 14 times the likelihood (OR95% CI, 13.94 (8.61, 2.59)) to be diagnosed as pulmonary tuberculosis comparing with those have not yet presented any symptoms (Table 2). This finding is consistent with previous studies but we could not determine whether the household contacts have been infected by TB cases inside or outside the family [1,2]. However, we find the prevalence of pulmonary tuberculosis and positive tuberculosis are respectively 20 times and 7 times higher than the notification rates which is publicly reported (Tables 1 & 2). This implies that early active case finding and treatment of tuberculosis patients without presenting symptoms among close contacts might bring benefit for the general public health. Although whether the screening strategy among the household contacts is cost-effective remains controversial, it is obvious that eradicating tuberculosis from the population totally needs to identify all potential transmission sources and eliminate them [17,18]. This concept of cost-effectiveness should be observed in a longer period of term and from a global perspective rather than measuring the cost and in local areas only. Furthermore, most Index Cases had a history of TB before being found and might contribute to drug resistance which will worsen TB burden. The on-going medical reform in China may increase the budget and expenditure on tuberculosis control [12]. Screening all household contacts of Index Cases is feasible and also a key step to bring tuberculosis control to sustainability in China. In fact, Index Cases are not always detected by passive surveillance now and increased numbers of tuberculosis patients have been detected by improved education and routine physical examinations [19].

In contrast to men, women are about 1.7 times more likely to transmit pulmonary tuberculosis to the household contacts while the male household contacts are more easily infected (AOR1.5) (Table 2). This may be due to the fact that women have more chance and longer time to be in contact with other family members. A local study and the national survey in rural areas in 2008 all indicate that about 74% of rural women’s work is responsible for taking care children and elderly people at home [20,21]. In the present study, more than 80% of Index Cases are in the rural areas, consistent with the distribution of tuberculosis in China [7,16].

The data from the present study do not support our hypothesis that cavitary tuberculosis, new smear positive cases and delayed reported patients may be associated with a higher probability of transmission within the home (Table 2). These findings may imply that Index Cases may not be the first patient in the family although they are the cases notified. Another unexpected outcome is the higher rate of non-tuberculosis cases in household contacts with symptoms (34/46 with symptoms vs. 37/46 without symptoms), which might be expected to be lower (<50%) in view of the low HIV prevalence in China. This unusual result might be due to wide mobilization during the study which may result in overstated symptoms when the household contacts were interviewed. Clearly further study is needed.

No bacterial genotyping was performed the present study. This disables us to identify whether the household contacts are infected by Index Cases or in the community. However, convincing evidence in the present study indicates that household contacts have more exposure to tuberculosis. There are a large number of potential pulmonary tuberculosis cases yet to be detected in China, irrespective of having symptoms or not. We also did not assessed HIV status for both Index Cases and the household contacts, which might influence our results, but China is still low HIV prevalence, which limits HIV/tuberculosis co-infection as a major confounder [22]. The type of building and ventilation of house might be associated with TB infection, but our study focused on rural areas where TB cases account for more than 80% in China. In these areas, there is little difference in the type of building and ventilation of house, so this factor will is not main contribution to difference in TB infection.

## Conclusion

Despite the limitation and controversies, the present study highlights that screening only the symptomatic contacts is clearly not enough to sustain the achievement of tuberculosis control in China, as well as in the world.

### Summary box-What is already known on this subject and what does this study add to? And policy implications

A few studies indicated that close contacts have a higher risk of developing active tuberculosis. China initiated a screening strategy for household contacts with suspicious symptoms of Index Cases in 2007 and more pulmonary tuberculosis patients have been notified than the routine surveillance. But national tuberculosis program has not included the screening among household contacts without symptoms due to inadequate resources in the past.

The present study examined the effectiveness of screening the household contacts without symptoms in 2008 and compared the effectiveness of screening household contacts with and without symptoms.

The on-going medical reform with mega health investment in China provides the promising opportunity for further improvement of tuberculosis case finding in China, including screening among household contacts without symptoms. The present study provides strong evidence to new tuberculosis screening guideline in China.

## Competing interests

The authors declare that they have no competing interests.

## Authors’ contribution

ZJ, SC designed this study, ZJ, SC, TZ and YM analyzed and explained the data, ZJ gave the first draft of the paper and ZJ, JZ and DC wrote final paper, LB, WX, XH and WZ contribute the data collection, all authors read the paper and contribute the work. All authors read and approved the final manuscript.

## Pre-publication history

The pre-publication history for this paper can be accessed here:

http://www.biomedcentral.com/1471-2334/14/64/prepub
